# Ising Ferromagnets on Proximity Graphs with Varying Disorder of the Node Placement

**DOI:** 10.1038/s41598-017-08531-8

**Published:** 2017-08-14

**Authors:** Hendrik Schawe, Christoph Norrenbrock, Alexander K. Hartmann

**Affiliations:** Institut für Physik, Universität Oldenburg, 26111 Oldenburg, Germany

## Abstract

We perform Monte Carlo simulations to determine the critical temperatures of Ising Ferromagnets (IFM) on different types of two-dimensional proximity graphs, in which the distribution of their underlying node sets has been changed systematically by means of a parameter σ. This allows us to interpolate between regular grids and proximity graphs based on complete random placement of nodes. Each edge of the planar proximity graphs carries a weighted ferromagnetic coupling. The coupling strengths are determined via the Euclidean distances between coupled spins. The simulations are carried out on graphs with *N* = 16^2^ to *N* = 128^2^ nodes utilising the Wolff cluster algorithm and parallel tempering method in a wide temperature range around the critical point to measure the Binder cumulant in order to obtain the critical temperature for different values of σ. Interestingly, the critical temperatures depend partially non-monotonously on the disorder parameter σ, corresponding to a non-monotonous change of the graph structure. For completeness, we further verify using finite-size scaling methods that the IFM on proximity graphs is for all values of the disorder in the same universality class as the IFM on the two-dimensional square lattice.

## Introduction

The Ising model of ferromagnetism^1^ is one of the most extensively studied models in statistical mechanics, because it features a continuous phase transition while being simple. The model consists of spins, which can be in one of two different states “+1” and “−1”. The pair-wise interactions of spins, can be expressed by graphs and thus there is a constant interest in the critical behaviour of this model on different graph ensembles. The research provides the whole range from analytical results for regular lattices^2, 3^ to numerical results for complex networks^4–7^. Further, there are many recent studies examining this model on different graphs^7–11^. This demonstrates the continuous interest in not only this model but also in related ones^12, 13^. While most of the time the behaviour of the model is in the focus of the research, one can also use the model as a measuring probe to find properties of the underlying networks^14, 15^.

An important type of graph is obtained from the Delaunay triangulation that finds application in, e.g., finite volume methods^16, 17^. For a system of randomly placed Ising spins on a two-dimensional surface with neighbour relationships derived from a Delaunay triangulation, the critical temperature has been obtained and it has been confirmed that it is in the same universality class, i.e. it shows the same critical exponents as the two-dimensional Ising model on a square lattice^18–20^ respectively in three dimensions on a cubic lattice^21^. This result is not trivial, since the Harris criterion suggests that random disorder is marginally important^22, 23^ for the two-dimensional Ising model.

The availability of results for this special case motivated us to extend and generalise the former research in two ways: First, we study two other types of irregular lattices, namely the *Relative Neighborhood graph* (RNG)^24^ and *Gabriel graph* (GG)^25^, which are subgraphs of the Delaunay triangulation^26^ and belong to the family of *proximity graphs*. The objective of proximity graphs is to connect nodes which are spatially close to each other, hence they are suited to generalise problems defined on regular lattices with nearest neighbour relationships. This family of graphs finds application in geographic variation studies in biology^27–29^, as potential candidates for “virtual backbones” in ad-hoc networks^30–34^ and in machine learning and pattern recognition^35^. Also, their statistical properties have been under scrutiny recently^36, 37^.

As a second generalisation and extension, we consider an ensemble of node sets depending on a tunable parameter *σ*. By changing this parameter we can interpolate the configurations between a square lattice and nodes distributed by a Poisson point process. A similar approach has recently been used^38^ to introduce an ensemble of Travelling Salesperson Problems (TSP) which interpolates between an easy to solve circular arrangement and a hard to solve random placement of the nodes. The increase of available computing power in recent years made it possible to perform the simulations at similar number of sites but increased number of realisations of the randomness and many values for *σ* in comparison to previous studies for the large *σ* limit^19, 20^.

For any node set the construction rule of a proximity graph defines the corresponding edge set. Thus, the parameter *σ* influences the regularity of the resulting proximity graph. Below, we measure the critical temperatures for a range of *σ*. While mean field theory *T*_c_ = *JK* and the Bethe approximation ${T}_{{\rm{c}}}=J/{\rm{a}}{\rm{t}}{\rm{a}}{\rm{n}}{\rm{h}}(\frac{1}{K-1})$ ^39^, which is practically linear for $K\mathop{ > }\limits_{\mathop{}\limits^{\eqsim }}3$, both suggest a linear dependence between *T*_c_ and the mean number of neighbours *K*, we find approximately a power-law relationship with an exponent which is small but certainly larger than one. Additionally, also to confirm the quality of our data, we verify by applying a common finite-size scaling analysis to obtain the critical exponents and by considering the two-point finite-size correlation function that the universality class does not depend on the graph type and does not change when varying *σ*.

The remainder of this work is organised as follows: First the RNG, GG and the Ising model are introduced. Then the details and results of our simulations are discussed. At the end a conclusion of this article is given.

## Model

### Proximity Graphs

An *undirected graph G*(*V*,*E*) consists of a set of nodes *V* and edges *E*⊂*V* ^(2)^. Two nodes connected by an edge are called *neighbours*. Proximity graphs are defined in a metric space. In this article a two-dimensional space with Euclidean metric is chosen, because it is the most common case and easy to visualise. Though every metric in any dimension can be used, in principle.

**Figure 1:**
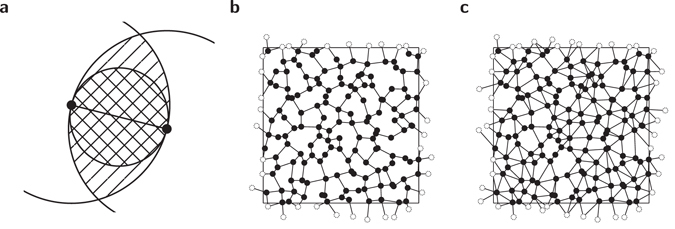
(**a**) Lunes of two nodes regarding the RNG (hatched region) and GG (cross hatched region), respectively. If there is no node located in the lune, both depicted nodes will be linked by an edge. (**b**) Example of the RNG with periodic boundary conditions. (**c**) Example of the GG with periodic boundary conditions. Mirror images of nodes (visualising the periodic boundary conditions) are shown in white.

One of the proximity graphs we consider here is the *Relative Neighbourhood graph* (RNG)^24^. Within this graph two nodes *i* and *j* with distance *d*_*ij*_ will be connected, if no other node is located in a well defined area called *lune*. The lune is defined as the intersection area of two disks with radius *r* = *d*_*ij*_, whose centres are on node *i* and *j*, respectively. This means that the edge {*i*, *j*} will be part of the RNG, if the condition

1 $${d}_{ij}\le \,{\rm{\max }}\{{d}_{ik},{d}_{kj}\}\quad \forall k\in V\backslash \{i,j\}$$

is fulfilled. For the sake of clarity, the lune of two nodes in regard to the RNG is sketched in Fig. 1a (hatched region). An example of the RNG is given in Fig. 1b. For a Poisson point process in a square, i.e. nodes placed on uniformly distributed, independent coordinates, its mean degree is *K* = 2.5576(3)^36^, i.e. the mean number of neighbours of each node.

The second proximity graph we study is the *Gabriel graph* (GG)^25^. In the GG two nodes *i* and *j* with distance *d*_*ij*_ will be connected, if the disk which has the connecting line between *i* and *j* as its diameter contains no further nodes *k*∈{*i*,*j*}, i.e. the condition

2 $${d}_{ij}^{2} < {d}_{ik}^{2}+{d}_{kj}^{2}\quad \forall k\in V\backslash \{i,j\}$$

is fulfilled. The lune is illustrated in Fig. 1a (cross hatched region). An example of the GG is given in Fig. 1c. Note that the lune of the GG is completely enclosed in the lune of the RNG, therefore the RNG is a subgraph of the GG and, as a consequence, all edges belonging to the RNG also belong to the GG. For a Poisson point process in a square, its mean degree is *K* = 4^37^–the same as the square lattice. Note that by construction RNG and GG have no crossing edges, i.e. they are *planar*. Furthermore they are *connected*, i.e. consist only of one connected component.

For any given set of nodes located in a two-dimensional plane these graphs can be constructed by an algorithm^36^ featuring a time complexity of ${\mathscr{O}}(N\,\mathrm{log}\,N)$. In this algorithm the Delaunay triangulation, which can be constructed in ${\mathscr{O}}(N\,\mathrm{log}\,N)$ ^40^ and which is a supergraph of the RNG and GG, is obtained first. Subsequently its edges, of which there are $|E|={\mathscr{O}}(N)$, are checked in regard to the aforementioned construction rules.

**Figure 2:**
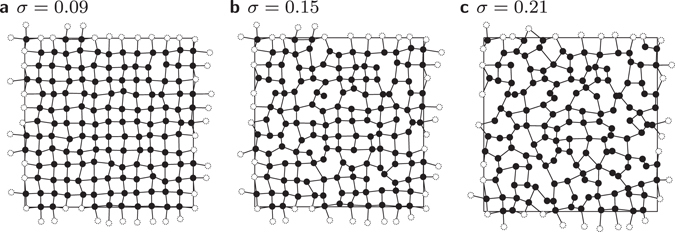
(**a**–**c**) Examples of proximity graphs (more precisely, RNG) for different values of *σ*. Connections which cross a periodic boundary are indicated by edges which connect a black node to a white node.

A simple construction of proximity graphs can be based on a uniform and random placement of nodes. To allow for a more complex behaviour, we apply a more general approach. Here, first the *N* = *L* ^2^ nodes are placed to form a regular square lattice with periodic boundary conditions. Next, the nodes are *displaced*, introducing geometric disorder resulting in a non-regular graph structure. The horizontal displacement Δ*x* and vertical displacement Δ*y* of each node is drawn from a Gaussian distribution with zero mean and standard deviation *σ*. Therefore, via the parameter *σ* we are able to interpolate between fully regular and fully random placement. The influence of *σ* on the structure of a proximity graph has two main effects which are clearly visible in Fig. 2 for the RNG. First, the number of edges changes as will be examined in more detail later on. Second, the variance of the length of the edges increases, i.e. there are some quite long and some very short edges in configurations with increasing *σ*.

### The Ising Model

Commonly, the IFM is studied on a square lattice with lateral length *L* and *N* = *L* ^2^ nodes with periodic boundary conditions. Each node *i* has an Ising spin *s*_*i*_∈{−1, +1} and interacts with its neighbours according to the Hamiltonian

3 $$ {\mathcal H} =-\sum _{\langle i,j\rangle }{J}_{ij}{s}_{i}{s}_{j}.$$

〈*i*, *j*〉 refers to nodes *i* and *j* wh*i*ch are nearest neighbours, i.e. they are connected by an edge. The coupling strength between *i* and *j* is g*i*ven by *J*_*ij*_.

In the generalised graph we use here, the interaction structure is not regular. To account for the varying distances, we choose the coupling constants *J*_*ij*_, which depend on the geometric distance between the connected pair of nodes. More precisely, the coupling constant of an edge {*i*, *j*} is

4 $${J}_{ij}={{\rm{e}}}^{\alpha \mathrm{(1}-{d}_{ij})},$$

where *d*_*ij*_ is the Euclidean distance between node *i* and *j*. Following the work of Lima *et al*.^20^. the free parameter *α* is set to *α* = 0.5. Note that distances smaller and larger than one can appear. For *σ* = 0 all *d*_*ij*_ = 1 and therefore all *J*_*ij*_ = 1 for every edge {*i*, *j*} ∈ *E*.

## Results

The results were obtained through Monte Carlo simulations at different temperatures. For each realisation of a graph, at each temperature independent configurations were simulated. For a *sweep* for all configurations *N* = *L* ^2^ single spin flip Metropolis attempts^41^ plus one Wolff cluster update^42^ was performed. To speed up equilibration, we allowed the configurations to change temperatures in a controlled way via applying the Metropolis-Coupled Markov Chain Monte Carlo (MCMCMC) method^43^, also known as Parallel Tempering^44, 45^. Thus, within one sweep an exchange of the configurations between a randomly chosen pair of neighbouring temperatures was attempted. Since it is not known beforehand, where the critical temperatures *T*_c_ are located, the Wolff cluster algorithm is used at every temperature. This combination of single-spin flip sweeps and Wolff steps guarantees for a *convenient* and rather fast equilibration and decorrelation at any temperature. Albeit the efficiency of the algorithmic procedure was not examined for every temperature, the speed up near criticality should be worth the moderate slow down at other temperatures.

For every system size *L* ∈ {16, 32, 64, 128} 19 different values of *σ* ∈ [0, 1.2] were considered. To average over the disorder, 100 different realisations are simulated for each system size *L* and disorder strength *σ*. Every realisation was simulated at about a hundred different temperatures *T* ∈ [0.1, 3.3] until at least 5000 independent measurements in equilibrium were obtained to get the thermal average, which needed up to 160000 sweeps.

### Reproduction of the Critical Exponents

First, we want to confirm that the Ising model on both planar proximity graphs belong to the same universality class as the Ising model on a square lattice. This can be since *dν* = 2 for the Ising model and therefore, according to the Harris criterion^22^, the disorder is not relevant, or at least border-line, i.e. its influence is hard to detect^46^. Therefore not only the critical exponents were obtained, but also a sensitive test using the two-point finite-size correlation function was done. While this examination will not yield any surprising results, it demonstrates the reliability of the simulation data and ensures the correctness of the simulations. The critical exponents were determined by a finite-size scaling (FSS) analysis^47^. Therefore we looked at the magnetisation *m*, susceptibility *χ* and Binder cumulant *g*, which are defined as

5 $$m=\frac{1}{N}\sum _{i}{s}_{i},$$

6 $$\chi =\frac{N}{T}{[\langle {m}^{2}\rangle -{\langle m\rangle }^{2}]}_{{\rm{avg}}},$$

7 $$g=\frac{3}{2}{[1-\frac{\langle {m}^{4}\rangle }{3{\langle {m}^{2}\rangle }^{2}}]}_{{\rm{avg}}}.$$

Here, 〈…〉 denotes the thermal average and […]_avg_ the average over the disorder realisations. For all quantities, we obtained statistical errors via bootstrap resampling^48, 49^.

**Figure 3:**
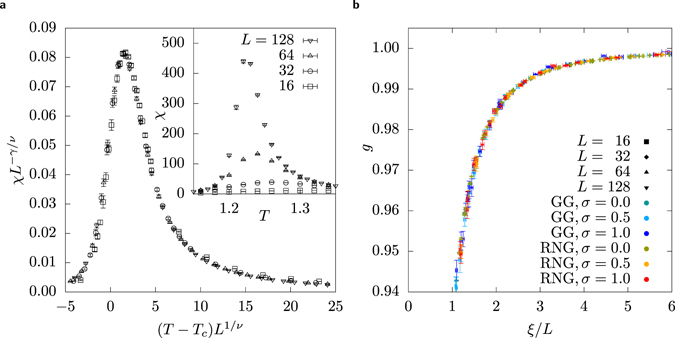
(**a**) Example of a data collapse of the susceptibility *χ* in regards to the RNG at *σ* = 1 to get the corresponding critical exponent *γ*. The same procedure is used on the Binder cumulant *g* and the magnetisation |*m*| to determine the critical exponents *ν* and *β* and the critical temperature *T*_c_ (see Table 1). The inset shows the same data with unscaled axes. (**b**) Binder cumulant *g* as a function of the two-point finite-size correlation function divided by system size *L*. The data points for different system sizes are drawn with different symbols, while the data points for different graph ensembles are drawn with different colours. All points fall on one curve within errorbars.

We determine the exponents by using the finite-size scaling assumption^50, 51^ for phase transitions of 2nd order, e.g., for the susceptibility *χ*:

8 $${\chi }_{L}={L}^{\gamma /\nu }\tilde{C}({L}^{\mathrm{1/}\nu }(T-{T}_{{\rm{c}}})),$$

where $\tilde{C}(\cdot )$ is some unknown scaling function. That means, for the right choice of *ν*, *γ* and *T*_c_ and appropriately scaled axes, the measurements at different system sizes *L* collapse on the scaling function as shown in Fig. 3a. A similar formula is valid for *m* (containing *β*) and *g* (which is dimensionless, i.e. has no prefactor in front of the scaling function). Values for the exponents and *T*_c_ are obtained in an automated and reproducible way^52^. They are visualised in Table 1 and are within errorbars consistent with the exactly known values for the square lattice IFM.

**Table 1:** Critical exponents for different values of *σ*.

	*σ*	*T*_c_	*ν*	*γ*	*β*
exact^58^	0	2.2691…	1	1.75	0.125
RNG	0.0	2.2688(7)	1.00(1)	1.74(1)	0.125(1)
	0.1	2.2053(6)	0.99(1)	1.75(1)	0.128(3)
	0.2	1.6265(19)	1.02(2)	1.76(1)	0.122(7)
	0.5	1.2825(9)	1.01(2)	1.75(2)	0.128(8)
	1.0	1.2125(5)	1.01(1)	1.76(1)	0.125(6)
GG	0.0	2.2688(6)	1.00(1)	1.74(2)	0.127(4)
	0.1	2.8944(43)	1.00(1)	1.75(1)	0.135(6)
	0.3	2.5281(27)	1.03(3)	1.72(2)	0.118(11)
	0.5	2.2388(9)	1.01(1)	1.75(1)	0.125(11)
	1.0	2.1275(20)	1.04(3)	1.75(2)	0.125(15)

A finite size scaling analysis was performed to determine the critical exponents *β*, *γ*, *ν* and the critical temperature *T*_c_. The errors for *β* and *γ* are estimated with the method from by an iterative method^52^. The errors of *ν* and *T*_c_ are the standard deviation of three obtained values through different collapses. The critical exponents are in reasonable agreement with the exact known values for the 2D Ising universality class. The critical temperatures *T*_c_ are shifting as expected.

A more sensitive method^53, 54^ to test whether two models are in the same universality class includes the analysis of the two-point finite-size correlation function

9 $$\xi =\frac{1}{2\,\sin (|{\overrightarrow{k}}_{{\rm{\min }}}|/2)}\sqrt{\frac{\chi (\overrightarrow{0})}{\chi ({\overrightarrow{k}}_{{\rm{\min }}})}-1}$$

where $\overrightarrow{k}$_min_ = (2*π*/*L*,0) and *χ* $\overrightarrow{k}$ is the wave vector dependent susceptibility

10 $$\chi (\overrightarrow{k})={[\langle |{(\frac{1}{N}\sum _{j}{s}_{j}\exp (ik{x}_{j}))}^{2}|\rangle ]}_{{\rm{avg}}},$$

which is in this case only evaluated over one direction, thus *x*_*j*_ is the position of the node in the horizontal direction.

We have plotted the Binder cumulant *g* as a function of *ξ*/*L* for different values of *σ*, *L* and *T* as shown in Fig. 3b. We can observe that within error bars all data points fall on the same curve, which confirms that the universality class does not change by varying *σ*.

### Behaviour of the Critical Temperature

**Figure 4:**
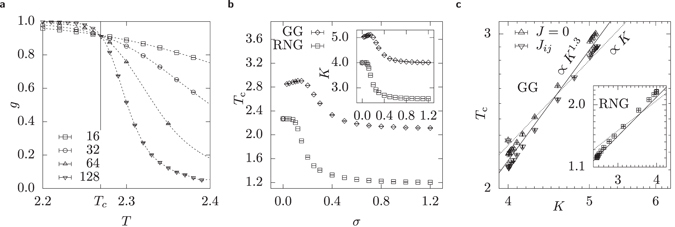
(**a**) Critical temperatures *T*_c_ are obtained by finding the intersections of the Binder cumulants *g* for different system sizes *L*. (**b**) *T*_c_ as a function of *σ*. The inset shows the average degree *K* as a function of *σ*. (**c**) *T*_c_ as a function of *K* on a log-log scale. The upper curve is for an ensemble where all edges have weight *J* = 1 and the lower curve for length dependent weight *J*_*ij*_. The inset shows the same for the RNG. It is clearly visible that the functional relation *T*_c_(*K*) is not linear (dotted line) as predicted by the Bethe approximation, but rather a power law *T*_c_ ∝ *K* ^*b*^ with an exponent of *b* ≈ 1.3 (solid line).

This study examines the critical temperatures *T*_c_ for different values of *σ*. In order to locate *T*_c_ we considered the Binder cumulants for different system sizes *L*, because they are intersecting at the critical point *T*_c_^55^ as shown in Fig. 4a. Since *g* is only measured for discrete values of *T*, the points have to be interpolated to find the intersection. Therefore a *cubic spline* ^56^ interpolation is calculated for the measured points. An even better method would be the *multiple histogram* method^45, 57^. But the simple cubic spline interpolation seems to deliver results that are good enough, since the comparison for the analytically known case *σ* = 0, i.e. square lattice, with *T*_c_ = 2.2691…^2^ suggests that the cubic splines offer good accuracy, since both ${T}_{{\rm{c}}}^{{\rm{RNG}}}\mathrm{(0)}=\mathrm{2.2690(2)}$ and ${T}_{{\rm{c}}}^{{\rm{GG}}}\mathrm{(0)}=\mathrm{2.2690(3)}$ are perfectly compatible with the reference value and offer reasonable precision.

**Figure 5:**
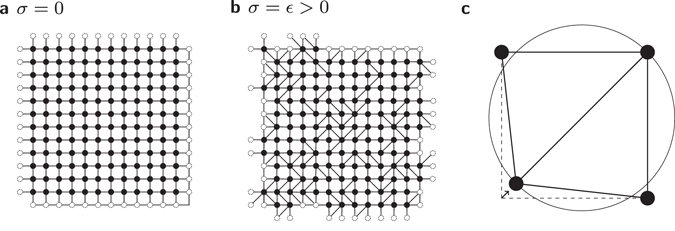
Visualisation of the occurence of the jump at small values of *σ* for the GG. (**a**) The regular lattice with no disorder. (**b**) Example of a graph for a very small value of the disorder. (**c**) Sketch of a GG with *N* = 4 nodes for very small *σ*. Due to the construction rule of the GG a new edge appears resulting in a jump in *K* and *T*_c_, see Fig. 4b. This does not occur in the RNG.

In Fig. 4b the critical temperatures are shown as a function of the disorder strength. Interestingly, we first observe that for the GG there occurs a jump from *T*_c_(0) = 2.2690(3) to a value close to the measured value *T*_c_(0.03) = 2.8511(7). This is easily explainable, because the square lattice at *σ* = 0 is a very special case for the GG. At very small deviations from the square lattice new edges arise in the GG, this is visualised in Fig. 5a–c. These new edges lead to a stronger coupling of spins such that the system stays ferromagnetic at higher temperatures. After an initial increase of the GG’s critical temperature with growing disorder, a maximum is reached and the critical temperature drops. Thus, the dependence is non-monotonous. This can be understood when analysing the dependence of the average number *K* of neighbours as a function of the disorder strength *σ*, as shown in Fig. 4b. The behaviour of *T*_c_ and *K* is very similar: *K*(*σ*) exhibits also a jump at *σ* = 0 as well as a peak close to *σ* = 0.2. This is confirmed when plotting *T*_c_ as a function of *K*, see Fig. 4c. While the Bethe approximation predicts an almost linear relationship *T*_c_ ∝ *K*, both proximity graphs show a power-law relationship *T*_c_ = *aK* ^*b*^ between the degree *K* and the critical temperature *T*_c_ with an exponent *b* ≈ 1.3. The same analysis is applied to a further simulation with all edge weights set to *J* = 1, i.e. *α* = 0, which also shows *b* ≈ 1.3. This can be interpreted as the edges chosen by the proximity graphs are more efficient to couple the spins than in the Bethe lattice. This stronger coupling is expressed by a higher *T*_c_. Note that the *σ* = 0 point is not part of this power law, which is expected, since it is a special corner case as mentioned above. Mind however, that the power-law relation is only approximately correct, since especially the RNG shows deviations from this form for low degrees *K*.

For large values of *σ* the node configurations approach the limit of a Poisson point process, hence *T*_c_ approaches a limit value, which was obtained from a fit as *T*_c_(*σ* = ∞) = 2.10(3) for the GG and *T*_c_(*σ* = ∞) = 1.19(1) for the RNG. A further interesting feature which highlights the importance of the structure of the graph for the critical temperature is the fact that the square lattice has a slightly different critical temperature than the GG in the Poisson point process limit although they share the same average degree *K* = 4^37^.

For the RNG, the critical temperature *T*_c_ of the RNG decreases smoothly and monotonously with increasing value *σ*. Therefore, the RNG exhibits neither a jump at *σ* = 0 nor a peak. This is reflected by the behaviour of *K*(*σ*) originating from a decrease of the number of edges, see Fig. 2 and Fig. 4b. Note also that for the RNG the power law between critical temperature and number of neighbours is well visible and also shows also an exponent of *b* ≈ 1.3 except for the smallest *K*. This is plotted in the inset of Fig. 4c.

## Conclusion

We have performed Monte Carlo simulations of an Ising Ferromagnet in a two-dimensional Euclidean plane with neighbour relationships defined by two different proximity graphs, the Relative Neighbourhood graph and Gabriel graph. Controlled by a disorder parameter *σ* we can smoothly interpolate between a regular square lattice node arrangement and a completely random node arrangement.

By means of finite-size scaling analysis and by studying the two-point finite-size correlation function, we confirmed the expectation that the universality class does not change for different graph types and for different values of *σ*. Furthermore, we have monitored the critical temperature *T*_c_ as a function of *σ*. For GG we observe a jump and a non-monotonous behaviour, reflecting non-trivial changes of the graph topology. On the other hand, for RNG, *T*_c_ stays approximately constant for small values of *σ* and decreases to some limit value for large disorder. In fact, *T*_c_ strongly depends on the degree *K*. It turns out that there is a power-law relationship between *T*_c_ and *K* for both proximity graphs. Even if the degree *K* = 4 of the GG at *σ* = 0 is equal to the degree for large values of *σ*, the critical temperature is lower at this point. Therefore, it becomes obvious that the average number of interaction partners is the dominating but not the sole influence for the location of the critical point.
